# The Lrp Family of Transcription Regulators in Archaea

**DOI:** 10.1155/2010/750457

**Published:** 2010-11-30

**Authors:** Eveline Peeters, Daniel Charlier

**Affiliations:** Erfelijkheidsleer en Microbiologie, Vrije Universiteit Brussel, Pleinlaan 2, 1050 Brussel, Belgium

## Abstract

Archaea possess a eukaryotic-type basal transcription apparatus that is regulated by bacteria-like transcription regulators. A universal and abundant family of transcription regulators are the bacterial/archaeal Lrp-like regulators. The Lrp family is one of the best studied regulator families in archaea, illustrated by investigations of proteins from the archaeal model organisms: *Sulfolobus*, *Pyrococcus*, *Methanocaldococcus*, and *Halobacterium*. These regulators are extremely versatile in their DNA-binding properties, response to effector molecules, and molecular regulatory mechanisms. Besides being involved in the regulation of the amino acid metabolism, they also regulate central metabolic processes. It appears that these regulatory proteins are also involved in large regulatory networks, because of hierarchical regulations and the possible combinatorial use of different Lrp-like proteins. Here, we discuss the recent developments in our understanding of this important class of regulators.

## 1. Introduction

The response and adaptation to environmental and nutritional changes, which is essential for the fitness and survival of microorganisms, is driven largely by regulation at the transcriptional level. In archaea, the vast majority of proteins that exert transcription regulation by binding the DNA and affecting gene expression are predicted to resemble bacterial classes of transcription regulators [1]. Almost 50% of all thus far identified regulators can be found in archaea and bacteria, while only 1.7% is common between archaea and eukaryotes [2]. Most of these predicted archaeal regulatory proteins possess a helix-turn-helix (HTH) DNA-binding motif, a typical bacterial motif. 

Intriguingly, archaea have a basal transcription machinery that is homologous to that of eukaryotes, albeit it being a simplified version, as is also the case for other information processes such as replication and translation [3–7]. Both *cis *and *trans *elements share homology with their eukaryotic counterparts. Cases in point are the main promoter elements TATA box and factor B recognition element (BRE) on the one hand, and the general transcription factors TATA-binding protein (TBP), transcription factor B (TFB), and the RNA polymerase (RNAP) on the other hand [3, 8]. The unique archaeal RNAP is most reminiscent of the eukaryotic RNAPII, having up to 13 subunits [9–11]. This peculiar hybrid situation raises the question as to how these bacterial-type regulators in archaeal organisms interact with the eukaryotic-like basal transcription machinery. This is especially true for regulators functioning as activators and exerting their regulatory effects by direct contacts with the general transcription factors [12] or RNAP, which are domain specific. 

Based on the analysis of genome sequences, it is predicted that the Leucine-responsive Regulatory Protein (Lrp) family, a well-known regulator family in bacteria, is also widespread and abundant in archaea [1, 2, 13–16]. The Lrp family, so named on the basis of the archetype Lrp that is a major global regulator found in *Escherichia coli*, is also referred to as AsnC (asparagine synthase C) family, named after another *E. coli* member, or Feast Famine Regulatory Protein (FFRP) family, referring to the general role of *E. coli *Lrp in the metabolic adaptation upon switches between nutritionally poor and rich media [17].

In bacteria, Lrp-like proteins constitute an important class of regulators since they are mostly involved in the regulation of the amino acid metabolism (biosynthesis, catabolism and transport) [18]. The widely studied global regulator *E. coli *Lrp regulates at least 10% of all genes, and new target genes are constantly being discovered [19–22]. This regulator can function as either a repressor or an activator, and this regulation can be in response to leucine, the main effector molecule, or effector-independent [17, 23, 24]. Consequently, six different regulatory strategies are being employed by Lrp. Generally, it shifts the amino acid metabolism between a “feast” regime (high nutritional availability), downregulating amino acid biosynthesis, and a “famine” regime (poor nutritional availability), stimulating amino acid biosynthesis [17]. Besides global regulators, many Lrp-like regulators that have been characterized are specific regulators, regulating the expression of only one or a few target genes or operons [18].

In archaea, although few researches on the function and structure of transcription regulators have been carried out, most of these studies focused on members of the universally present Lrp family. Although research is limited to case studies of Lrp members in the model organisms *Pyrococcus*, *Sulfolobus*, *Methanocaldococcus*, and *Halobacterium*, information is becoming available on different aspects of these regulators: structure, DNA binding, effector binding, and physiological function. At present time, they can be considered as one of the best studied families of regulators in archaea. However, there is still a great lack of knowledge on target genes and effector molecules of these regulatory proteins. 

## 2. Phylogenetic Distribution

The prediction of genes encoding Lrp-like transcription regulators is not straightforward because of their relatively low sequence conservation (with an average amino acid sequence identity of between 20% and 30%). Nevertheless, multiple efforts to predict genes coding for these proteins in archaeal genomes, mainly by hidden Markov model-based searches, have revealed a wide phylogenetic distribution [1, 2, 13–16, 18, 25]. The presence of this regulator family in both archaea and bacteria suggests that the last universal common ancestor already possessed a prototype of Lrp-type regulators [18, 26]. A loss early in the eukaryal lineage could explain its absence in eukaryotes [18], although the acquisition by horizontal gene transfer cannot be excluded. The ancestral nature of this type of regulators emphasizes their involvement in the regulation of core-metabolic functions, of which some were already present in the last universal common ancestor.

Lrp is one of the three most abundant transcription regulator families in archaea (besides ArsR and HTH_3) and corresponds to about 8% of all non-general transcription factors identified in 52 archaeal genomes [2]. Full-length Lrp-like proteins are universally present in all archaeal genomes sequenced to date, either belonging to Euryarchaeota, Crenarchaeota, Korarchaeota, or Nanoarchaeota [2]. Most archaeal Lrp-like regulators resemble more closely the bacterial member AsnC, a specific regulator of asparagine biosynthesis found in *E. coli*, than the well-known global regulator *E. coli *Lrp [26]. There is a correlation between the total number of transcription regulators and the adaptability of the organism to changes in the nutritional conditions [2]. The number of *lrp*-like genes in archaeal genomes, as predicted in a thorough genomic analysis of transcription factors [2], seems to be proportional to the total number of regulators. Therefore, a similar correlation between the lifestyle and the repertoire of Lrp-like regulators can be assumed. Limited sets of Lrps can be found in species that are confined to habitats with very specific nutritional requirements, such are the cases of methanogens that are mostly autotrophic. The same accounts for organisms with a restricted metabolism, which exhibit a limited or no *de novo *amino acid biosynthesis and that are thus dependent on an external supply of amino acids. Here, one of the most extreme cases is the symbiotic *Nanoarchaeum equitans* [27], which is predicted to contain only two regulators belonging to the Lrp family [2]. On the other hand, archaea with a high metabolic diversity that are able to grow heterotrophically on a wide variety of different substrates, usually contain a large repertoire of these regulatory proteins. 

## 3. Three-Dimensional Structure

**Figure 1:**
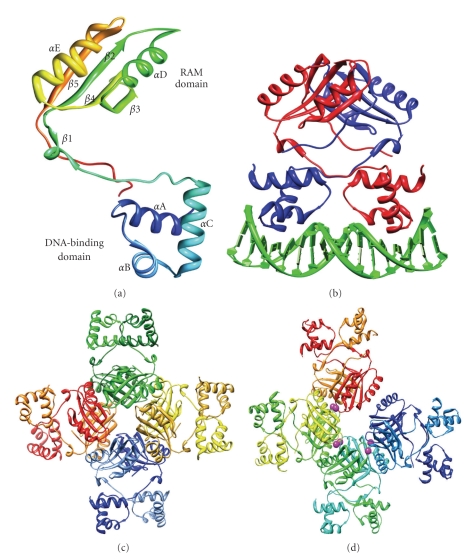
Structural features of archaeal Lrp-like proteins. (a) Monomeric structure of Grp from *S. tokodaii* (PDB 2E7W) [31]. Secondary structure elements are named as follows: *α*-helices are called *α*A–*α*E and *β*-strands *β*1–*β*5. The N-terminal DNA-binding domain corresponds to the bottom part of the structure, the C-terminal ligand binding domain to the top part. These are labeled DNA-binding domain and RAM domain, respectively. (b) Cocrystal structure of an FL11 dimer bound to DNA (PDB 2E1C) [30]. (c) Octameric structure of LrpA from *P. furiosus *(PDB 1I1G) [28]. (d) Cocrystal structure of an arginine-bound octamer of FL11 from *P. horikoshii*, which has an open conformation (PDB 2ZNY) [32]. The position of the arginine molecules is shown by purple symbols.

Despite their low sequence identities, Lrp-like proteins have a highly conserved structure. Crystal structures of several archaeal Lrp members have been determined, either in the apo form, or in the holo form bound to their respective effectors (Figure 1) [28–32]. In fact, LrpA from *Pyrococcus furiosus* was the first Lrp-like protein to have its structure characterized [28]. The observation that most archaeal Lrp-like proteins crystallize as octamers or higher association forms demonstrates the ability of these proteins to oligomerize. This is most clearly illustrated by the *Pyrococcus horikoshii *protein FL11, of which the apo form crystallized into right-handed helical cylinders, extending from one side of the crystal to the other [29]. 

Lrp-like proteins are typically 15 kDa monomers in which two domains are defined: the N-terminal DNA-binding domain, containing a HTH motif, and a C-terminal domain that facilitates oligomerization and effector binding (Figure 1(a)). This motif is a winged HTH, which is the most abundant DNA-binding motif found in archaea [2]. The C-terminal part is also called Regulation of Amino acid Metabolism (RAM) domain [33]. These two domains are connected through a hinge of approximately 15 amino acids in length and containing one *β* strand (*β*1). In all known crystal structures this linker is rather well structured, demonstrating a conformational rigidity to a certain extent.

The C-terminal RAM domain, which adopts an *α* *β* sandwich fold having an antiparallel *β* sheet composed of 4 strands “sandwiched” between 2*α* helices, is responsible for oligomerization (Figure 1(a)). The minimal functional structural unit is a dimer that is formed by an interaction between different *β* strands, *β*2, and *β*5, belonging to the two RAM domains and establishing a stable hydrophobic core (Figure 1(b)). In solution, archaeal Lrp-type regulatory proteins form not only dimers, but also frequently multimers of dimers: tetramers, hexamers, octamers, or dodecamers [28, 29, 34–40]. These higher association states are achieved through further interactions between the C-terminal cores of dimeric forms, involving the *β*5 and *α* chains (Figure 1(c)). The resulting higher-order assembly exhibits a central core of interacting RAM domains and the DNA-binding domains facing outwards. Generally, an oligomeric heterogeneity is observed, in which the relative concentration of each oligomeric species is influenced by factors such as pH, protein concentration, DNA binding, or the binding of specific effector molecules [28–30, 41, 42]. This complex dynamic equilibrium of different oligomeric states is one of the key determinants of the regulatory mechanisms of Lrp-like proteins and the way they respond to their effectors. Several archaeal stand-alone RAM protein structures, lacking a DNA-binding domain, have been reported as well [41–43]. As yet, their exact physiological significance remains unclear.

## 4. Binding of Effector Molecules

Small molecules, representing nutritional or metabolic “signals”, bind the Lrp-like regulatory proteins and induce regulatory effects. To date, all small molecules that bind Lrp-like regulators are amino acids (Table 1) [30, 31, 38, 41, 42, 44, 45]. Most regulators, of which the function is characterized, are involved in regulating amino acid metabolism. This suggests that variations in cellular amino acid concentrations lead to a direct and fast response in gene expression. The amino acid molecules that interact with an Lrp-like regulator can either result from *de novo* biosynthesis or originate from the environment after uptake by the cell, which is generally an energetically more economical process. Ligand specificity can vary notably. Some regulators have a broad amino acid specificity range while others are restricted to interact with only one type of amino acid (Table 1). In contrast with bacterial Lrp-like regulators, for which only amino acids have been identified as effectors, it appears that some archaeal Lrp-like proteins might interact with other small molecules. The indications for this are that some of the regulators are also involved in other metabolic processes apart from amino acid metabolism and that sequence conservation in the ligand-binding pocket of certain archaeal proteins is very low as compared to proteins known to bind amino acids. None of the twenty amino acids have an effect on DNA binding by Ss-LrpB from *Sulfolobus solfataricus *and by the regulator encoded by ST1115 from *Sulfolobus tokodaii *(unpublished observations from our laboratory). Although not experimentally proven, it is also predicted that LrpA from *P. furiosus*, Ptr2 from *Methanocaldococcus jannaschii*, and several Lrp-like regulators from *P. horikoshii* do not interact with amino acids [26, 28, 46]. Furthermore, the stand-alone RAM domain protein DM1 has been reported to respond also to other metabolic intermediates besides amino acids, such as 2-oxoglutarate [25].

The binding pocket is located in the RAM domain of the protein, formed by *β* strands and loops originating from separate dimers (Figure 1(d)) [25, 41]. For amino acid interacting regulators, the residues in the RAM domain that interact with the backbone of the amino acid are well conserved while the residues that interact with the specific side chain are poorly conserved [42]. By analyzing the contacts made by these residues in the holo structures, it was possible to deduce a “structural code” that was used to predict the effector amino acids of novel Lrp-like proteins [41]. The prediction that Sa-Lrp from *Sulfolobus acidocaldarius *interacts with glutamine was experimentally confirmed (unpublished observation from our laboratory). Generally, at each dimer-dimer interface two amino acid molecules bind, likely by diffusing into the assembly through the central cavity inside the four interacting dimers [25, 29]. Curiously, an exception to this quite consistent pattern is observed for Grp from *S. tokodaii* [31]. Here, besides the typical binding site in the RAM domain, a second binding site for the effector molecule, in this case glutamine, was identified at the *α*C helix of the HTH motif. 

Effector binding can lead to different types of structural changes that convert the signal into a regulatory response. Three categories are observed: (i) very subtle conformational modifications restricted to the neighbourhood of the binding pocket [31], (ii) large conformational changes; an example is the binding of arginine to FL11, which induces the octamer to adopt an open conformation (Figure 1(d)) [32], (iii) modulation of the oligomeric state of the protein. The latter is reminiscent of the effect of leucine on the global Lrp regulator in *E. coli*, where it is well established that leucine induces the dissociation from hexadecamers to leucine-bound octamers [47]. Effector binding can either promote association or dissociation of the multimers [25, 30, 41, 42, 46]. An association shift, usually from dimers to octamers, appears to be more common, given that the ligand binding sites are located at the dimer-dimer interfaces and that in certain cases this could stabilize interdimer interactions. In the case of regulators with a broad effector specificity, effector-induced conformational changes in a protein can also be differential depending on the type of ligand. A case in point is FL11: a closed fourfold symmetrical octamer is formed upon binding of lysine, whereas upon addition of arginine, an octamer with an open conformation is observed (Figure 1(d)) [32]. Another example is the case of the stand-alone RAM domain protein DM1 in *P. horikoshii* where isoleucine promotes association to octamers, while methionine generates the opposite effect and stabilizes dimers [41, 48].

## 5. DNA-Binding Properties

The minimal unit of an Lrp-type protein able to interact with the DNA, is the dimeric form. As is the case for the bacterial members, archaeal Lrp dimers generally recognize 13- to 17-base pair long sites with an imperfect inverted repeat [36, 49, 50]. Each site contains an AT-rich centre with the minor groove facing towards the interacting dimer and two half sites, each contacted in the major groove by the recognition helix *α*C, as is typical for the HTH motif [51, 52]. The only known cocrystal structure of an Lrp-like regulator bound to a DNA fragment, is that of the archaeal FL11 dimer bound to a fragment containing a 13 base pair palindromic site (Figure 1(b)) [30]. As seen in the cocrystal structure, residues in the loop between *α*B and *α*C and a few residues from *α*B are involved in the interaction as well, besides the recognition helix (Figure 1(b)) [30]. Among other residues, several arginines that are present in the recognition helix are important for contacts in the major groove of the DNA [30, 49]. One of these arginines is located at a highly conserved position, and its substitution by an alanine results in the complete abolishment of binding [35, 36]. 

Several attempts have been undertaken to characterize the DNA-binding specificity and to define a consensus binding site sequence for different characterized archaeal Lrp-like proteins. This was done either by (i) SELEX approaches, allowing selection of the best binding sites out of a large set of artificially made random sites or of genomic sequences [36, 50, 53, 54] or by (ii) analyzing binding to a set of saturation mutants starting from a consensus site based on known binding sites [49]. The advantage of the latter method, which was applied to the *S. solfataricus *member Ss-LrpB, is that it reveals the entire energy landscape of binding. The DNA-binding sequence specificities of Lrp-like regulators can vary widely and the general trend is that the natural binding sites are quite degenerated. Therefore, it is generally not easy to predict the location of potential binding sites in the genome sequence based on the already known binding specificity [36]. 

*In vitro*, DNA binding is often studied as a simple binding event of a dimeric Lrp-like protein to a site with a (semi-) palindromic sequence. However, in the genome, Lrp target regions always seem to occur as clusters of multiple sites that are imperfect to variable degrees. Different patterns of binding can be discerned: (i) cooperative binding of multiple binding sites by separate dimers, whereby these dimers closely interact and might form a DNA-induced higher oligomeric form [39, 46, 51], (ii) binding of a higher oligomeric form, for example, a tetramer, to one or multiple regularly spaced well-aligned binding sites [12, 34, 38, 55], (iii) less specific binding of higher protein assemblies to large regions of DNA (generally more than 100 base pairs), in which only highly degenerated recognition sites can be recognized [35, 37, 56]. The alignment between adjacent binding sites is governed by the helical periodicity, accommodating binding of the different dimeric units to the same face of the DNA helix. A centre-to-centre spacing of either two or three helical turns has been observed [12, 46, 51, 57]. In some cases, different patterns of binding can be observed for the same Lrp-type regulator, possibly depending on oligomeric changes, which in turn might be affected by effector binding. Such examples are (i) Ptr2, that protects a long stretch of DNA in the control region of its own gene but binds two regularly spaced sites in the control regions of its targets [12, 36], and (ii) FL11, of which the lysine-bound octamer binds a long stretch of DNA, while the dimeric apo form binds to individually recognizable binding sites [30, 41]. The more common bacterial strategy, in which effector-induced conformational changes lead to either a decrease or an increase of the DNA-binding affinity, is also observed for LysM from *S. solfataricus *and Grp from *S. tokodaii* [31, 38]. 

Lrp-like regulators may induce strong conformational changes in the DNA structure upon binding. Binding of an archaeal Lrp-like dimer to a single site results in bending of the DNA with an angle of about 50° [30, 51], while interaction with a higher oligomer, or multiple interacting dimers, causes the DNA to wrap around the protein. This wrapping is assumed to occur based on the structural characteristics of the protein (peripheral DNA-binding domains) and on the observation of hyperreactive or hypersensitive zones in footprinting experiments [36, 46, 51, 55, 56]. Furthermore, these types of higher order nucleoprotein complexes have been visualized with microscopy techniques for *S. solfataricus *Ss-LrpB and *P. horikoshii *FL11 [29, 39, 58]. The first forms complexes with about 100 base pairs of DNA wrapped around three interacting dimers, while the latter induces a positive supercoil by interacting as an octamer [30]. The energetic cost of inducing these large conformational changes in the DNA, is assumed to be compensated by favorable protein-DNA interactions and, in the case of cooperative binding, protein-protein interactions [36].

## 6. Molecular Mechanisms of Regulation

Modulation of gene expression is usually achieved by interaction with the preinitiation complex of transcription. Regulatory effects can be studied by *in vitro *transcription experiments using a re-constituted archaeal transcription system containing TBP, TFB, RNAP and varying amounts of the Lrp-like regulator [12, 30, 34, 55, 57]. To present date, only two regulation mechanisms have been unraveled at the molecular level [12, 55]. Generally, archaeal Lrp-like regulators bind close to the promoter region, allowing the formation of a ternary TBP-TFB-Lrp complex, but affecting the formation of the preinitiation complex. This effect can be either positive or negative, while yet others are dual regulators able to switch between activator and repressor functions. Based on the existence of different DNA binding patterns and locations, it appears that a large variation of regulation strategies might be employed, but these mechanisms are not yet well understood. For instance, a possible scenario is that Lrp-induced DNA wrapping, which changes the local topology of the DNA, also affects the transcription efficiency, perhaps in postrecruitment steps of transcription initiation.

Repression is demonstrated for LrpA from *P. furiosus* to take place by inhibiting the recruitment of RNAP [55]. LrpA is able to bind its own gene's control region simultaneously with TBP and TFB by interacting with a region that borders the TATA box at its downstream side. However, RNAP cannot bind the preinitiation complex while LrpA is associated through steric hindrance. This type of regulation allows a fast response to environmental changes given that upon release of the regulator, only RNAP needs to bind before transcription is initiated. This molecular mechanism of regulation was previously observed for a metal-dependent transcription regulator MDR1 from *Archaeoglobus fulgidus *[59]. An alternative repression mechanism for archaeal regulators, in which the entire promoter region is occluded, thereby inhibiting the first step in the formation of the preinitiation complex (binding of TBP and TFB), is proposed to exist for Lrp-type regulators as well, but this has not yet been experimentally proven [30, 35, 37]. Lrs14 from *S. solfataricus* employs this mechanism in its negative autoregulation but this protein, although annotated originally as Lrp-like, is now predicted not to belong to the Lrp family [2, 60]. 

For the archaeal Lrp-like proteins Ptr2, LysM, and Ss-LrpB, it has been shown (or there are strong indications) that they function as an activator [12, 38, 46, 57]. A common observation is the presence of a dimer-binding site just upstream of the promoter, separated by only a few base pairs from the BRE element. The position of this binding site relative to the promoter is highly constrained [57]. Activation is exerted by the dimer bound to this site (whether this dimer is part of a larger oligomeric assembly or not) through direct protein-protein interactions with TBP, thus leading to a stimulated recruitment, and probably also by affecting postrecruitment steps [12, 40, 57]. Additional low- or high- affinity binding sites can be present upstream of this core site responsible for activation, and these are also important in the context of the regulatory mechanism [46, 51, 57]. This could be explained by (i) facilitating the binding of a higher oligomeric form (Ptr2) or by (ii) providing a means to form higher-order protein-DNA complexes through cooperative binding which influences the regulatory effects (Ss-LrpB; see below). Possibly, other operator organizations could also result in activation: for two activated target genes, Ss-LrpB binding occurs at a core binding site more than 100 base pairs upstream of the transcription start and only extends further downstream at higher protein concentrations [46]. In the RAM domain, several surface-exposed amino acids have been identified to be involved in activation, which is being hampered after mutating these residues [40, 61]. Inversely, mutating residues in TBP also negatively affected activation by Ptr2 [62]. Given the fundamental differences between the bacterial and archaeal basal transcription machineries, it can be assumed that in the archaeal lineage of the Lrp family, the regulators have evolved differently to be able to interact with the eukarya-like TBP.

Archaeal Lrp-like regulators are very diverse, and for some it has been shown that they can function both as a repressor and an activator, depending on the location of the binding site, the architecture of the Lrp-DNA complex and/or the oligomeric form of the protein [12, 44]. Ss-LrpB functions as a dual regulator on the promoter of its own gene in a concentration-dependent manner (see [39, 51], unpublished results). The regulator acts as a genetic “switch” between positive autoregulation at low protein concentrations and negative autoregulation at higher concentrations. Cooperative binding of dimers and DNA wrapping are key components of this switch mechanism. Another example is *M. jannaschii *Ptr2 of which the open reading frame overlaps the control region of its target [12]. Here, it is a possibility that Ptr2 functions as a roadblock, affecting the transcription of its own gene at a later stage than initiation in a negative sense, while modulating transcription initiation of the targets positively at the same time. Finally, effectors can have varying roles in the regulatory mechanism, and are usually co-repressors, amplifying repression (FL11) or inactivating activation as proposed for LysM [30, 38].

## 7. Physiological Functions

For most Lrp-like regulators in archaea, the physiological role and the identity of the target genes remains to be elucidated. One possible approach to find these targets is to construct a mutant strain having the regulatory gene deleted. However, until recent developments, genetic techniques were difficult to apply in archaea, especially in hyperthermophiles. Only in *S. solfataricus* and in *H. salinarum*, *lrp *deletion strains have been constructed allowing an easier assignment of target genes [44, 46]. Often, the *lrp *genes are located in the direct vicinity of their target genes, either transcribed as a polycistronic messenger with its target (as observed for *lrpA1* homologs in halophiles [44]) or transcribed in separate units. Different organizations have been observed: regulatory gene and target gene(s) can be organized divergently or convergently, having common or separate control regions [12, 38, 44, 46]. 

In comparison to bacterial Lrp members, the archaeal ones are not as restricted to regulating amino acid metabolism and have more versatile functions (Table 2) [18]. It appears that not only the global Lrp-type regulators, but also those with more narrow regulatory actions, are responsible for the regulation of genes involved in energy and central metabolism and transport as well (Table 2) [12, 26, 44, 46, 57]. For Ss-LrpB from *S. solfataricus*, deletion analysis has shown that the regulator activates a pyruvate ferredoxin oxidoreductase operon and two permease genes [46]. Other Lrp-like regulators, belonging to different archaeal lineages, also regulate genes encoding subunits of the class of 2-oxoacid:ferredoxin oxidoreductases, universally present in archaea and involved in catalyzing different central metabolic reactions [63]. The Ptr2 target ferredoxin shows similarity with one of the subunits of one of these enzymes [26, 46]. Several *Thermoplasma volcanium *Lrp-like regulators are predicted to be involved in the transition between aerobic and anaerobic growth, possibly sensing oxygen levels [26, 64]. The suggestion that some regulators respond to other metabolites than amino acids, also indicates their versatility.

As in bacteria, some Lrp-like regulators have a specific, local role, while others have a global role (Table 2). So far, two global Lrp-type regulators have been characterized in archaea: FL11 from *P. horikoshii* and Lrp from *H. salinarum*. FL11 is predicted to regulate about 200 transcription units in response to lysine. In certain aspects, this master regulator appears to be reminiscent of the global regulator Lrp in *E. coli*. The cellular amount of both regulatory proteins is dependent on the growth phase and quite abundant [30, 65]. Furthermore, FL11 can also be considered as a “feast or famine regulator” coordinating cellular states of growth or rest. In the first regime, amino acid catabolism and ATP synthesis is derepressed while in the second regime, amino acid biosynthesis genes are derepressed and the cells stop growing [30]. The *Halobacterium *global regulator Lrp influences not only genes involved in amino acid metabolism and central metabolic pathways, but also peptide and phosphate transporters [44]. The effector molecule(s) of this regulator are, as yet, unknown.

Finally, it is interesting to note that some of the regulators modulate the expression of other transcription regulators, whether or not these belong to the Lrp family, indicating the existence of hierarchical regulatory networks [26, 44]. The global regulator FL11 in *P. horikoshii *is predicted to influence the expression of at least four other Lrp-type regulators. The hierarchical buildup seems to be dependent on the metabolic importance of the respective effectors and on the radius of their regulatory action; that is, whether they are global or specific regulators. Two characterized *Halobacterium *regulators are also shown to regulate *tfb *genes [44]. This is striking since *H. salinarum *contains multiple copies of *tbp* and *tfb *genes, and it is believed that they are involved in regulating different sets of promoters reminiscent of the use of alternative sigma factors in bacteria [66–68]. Therefore, there is a link between this type of global gene regulation and the control by the Lrp-type regulators. Most Lrp members also exert an autoregulation, effector-dependently or -indepently, which is yet an additional level of control [29, 31, 34, 36, 44, 51]. 

## 8. Concluding Remarks

Genes predicted to encode transcription factors in archaeal genomes constitute on average less than 5% of all gene products [2]. This is low in comparison to bacteria. However, a significant portion of these regulators belong to the Lrp family, with most archaea having 5 to 10 or even more different Lrp regulators. Lrp-like regulators seem to play an important role in coordinating cellular archaeal metabolism in response to environmental alterations, and they may therefore contribute to the fitness of the cells. We are only at the beginning of understanding the enormous complexity of this important family of regulators. On the basis of the limited information that is available, it is possible to speculate about their roles in archaeal gene expression. 

There seems to be a vast repertoire of different regulatory strategies, determined by association and dissociation in different oligomeric assemblies, different patterns of DNA binding, different regulatory mechanisms, and different spectra and specificities of effectors and strategies to respond to them. The combination of these elements leads to an impressive plasticity of gene regulation. Furthermore, different Lrp-like regulators in an archaeon are probably involved in networks. Instead of an independent occurrence of distinct one-to-one regulator-target combinations, regulatory events seem to be intertwined. Some Lrp-like regulators seem to regulate the expression of other Lrp-like regulators, according to a hierarchy, and are most probably also able to form heteroassemblies, in accordance with eukaryotic regulators, for example, heterodimeric leucine zippers [69]. The formation of a heterooctamer has been shown to exist *in vitro* for the full-length FL11 and the stand-alone RAM domain protein DM1 of *P. horikoshii *[41]. It could be imagined that each of these heteroassemblies, made up of dimeric units of different Lrp-like regulators, has different effector responses, DNA-binding and regulation characteristics. Therefore, this combinatorial use may lead to an exponential increase in strategies to modulate gene expression and to a less strict classification of global or specific regulators. The involvement of stand-alone RAM domain proteins in hetero-oligomers, also provides a possible explanation for their existence.

## Figures and Tables

**Table 1:** Archaeal Lrp-like regulators with identified effector molecules. Generally, effector molecules have been identified based on effects on association state or DNA binding. This does not imply that they have a regulatory function. Stand-alone RAM domain proteins are indicated with an asterisk.

Organism	Name	Effector(s)	Reference
*H. salinarum *	LrpA1	Asp	[44]
*P. horikoshii*	DM1*	Ile, Val, Arg, Leu, Met, Phe	[41]
*P. horikoshii*	DM2*	Gln	[41]
*P. horikoshii*	DM3*	Phe, Val, Met, Ile, Leu	[41]
*P. horikoshii*	FL4	Glu	[41]
*P. horikoshii*	FL5	Phe, Ile, Leu, Val, Met	[41]
*P. horikoshii*	FL11	Lys, Arg, Gln	[30]
*S. solfataricus *	LysM	Lys	[38]
*S. tokodaii*	Grp	Gln	[31]
*S. tokodaii *	STS042*	Ile	[42]
*T. volcanium*	TvDM*	Ile, Leu, Phe, Met, Val	[41]
*T. volcanium*	TvFL3	Lys	[41]

**Table 2:** Archaeal Lrp-like regulators with identified target genes or operons. Autoregulatory targets and targets with unknown function are not included in this list. For FL11, only a fraction of the (potential) targets are shown [30].

Organism	Name	Target(s)	Biological process	Reference
*H. salinarum*	Lrp	*tfbF*	transcription	[44]
		*pstC2*	transport	
		*phnC*		
		*dppF1*		
		*dppD1*		
		*dppC1*		
		*dppB2*		
		*glnA*	amino acid metabolism	
		*korB*	central metabolism	
		*korA*		
		*gldA1*		
		*car*	signal transduction	

*H. salinarum*	LrpA1	*aspB3*	amino acid metabolism	[44]
		*tfbB*	transcription	

*M. jannaschii*	Ptr2	*fdxA*	electron transport	[12, 57]
		*rb2*		
		*rbr*		

*P. furiosus*	LrpA	*vor*	central metabolism	[25]
		*por*		

*P. horikoshii*	FL11	ATPase	ATP biosynthesis	[30]
		NAD(P)H dehydrogenase	central metabolism	
		*lysJKYZ*	amino acid metabolism	
		*leuABCD*		

*S. solfataricus*	LysM	*lysWXJK*	amino acid metabolism	[38]

*S. solfataricus*	Ss-LrpB	*porDAB*	central metabolism	[46]
		permeases	transport	
